# Household determinants of delayed MMR vaccination: longitudinal analysis using electronic health records in North East London, UK

**DOI:** 10.1136/bmjopen-2024-097559

**Published:** 2025-05-02

**Authors:** Milena Marszalek, Nicola Firman, Marta Wilk, Ana Gutierrez, Kelvin Smith, Carol Dezateux

**Affiliations:** 1 Centre for Primary Care, Wolfson Institute of Population Health, Faculty of Medicine and Dentistry, Queen Mary University of London, London, UK

**Keywords:** Child, Public health, EPIDEMIOLOGY, Primary Care, Paediatric infectious disease & immunisation

## Abstract

**Abstract:**

**Objectives:**

There is a lack of information about household factors associated with delayed measles, mumps and rubella (MMR) vaccination. We examined whether timeliness of first MMR (MMR1) receipt is associated with sharing a household with an older child with non-receipt of MMR1 independent of household composition and size.

**Design:**

Longitudinal observational study using linked electronic health records.

**Setting:**

North East London, UK.

**Participants:**

The index cohort comprised 71 509 children (51.0% males) eligible to receive MMR1 between 1 January 2014 and 28 February 2020.

**Methods:**

The primary outcome was MMR1 receipt between 12 months and 24 months of age. The explanatory variable was non-receipt of MMR1 between age 12 months and 24 months in the oldest child sharing the same household. We examined the likelihood of MMR1 receipt in index children sharing a household with an older child with non-receipt of MMR1 between 12 months and 24 months using logistic regression to estimate ORs and 95% CIs before and after adjustment for individual-level, household-level and area-level covariates. We carried out sensitivity analyses excluding households with an age interval between oldest and youngest child greater than 5 years.

**Results:**

59 851 (83.6%) index children received MMR1 between 12 months and 24 months of age. After adjustment for household composition and size, MMR1 receipt was less likely in index children sharing a household with an older child with non-receipt of MMR1 between 12 months and 24 months of age: OR: 0.19 (95% CI: 0.18, 0.20). This association strengthened after excluding households with an age interval greater than 5 years: OR: 0.14 (0.13, 0.15).

**Conclusions:**

There is strong concordance within households of delay in MMR1 receipt independent of household size and composition. Lack of timely protection within households increases the risk of measles outbreaks. There is a need for household-based interventions to improve MMR1 timeliness.

**STRENGTHS AND LIMITATIONS OF THIS STUDY**

- We used a novel method to link individuals into households while maintaining privacy and confidentiality using electronic health records (EHRs) for a large population.
- We obtained high-quality, accurately coded and validated measles, mumps and rubella data in the EHR.
- We used robust statistical methods to assess relationships between the exposure and outcome variables.
- Processes of and influences on decision-making about vaccines between the linked younger and older children may have differed. We were not able to examine associations with delayed receipt of primary vaccinations against diphtheria, pertussis, polio, tetanus and *Haemophilus influenzae*.
- More granular categorisation of ethnic groups, as suggested by our patient and public involvement group, was not possible due to limited sample size.

## Introduction

 Childhood vaccinations form an essential part of public health interventions provided by primary care.^1^ In England and Wales, it is recommended that children receive a first dose of measles, mumps and rubella (MMR) vaccine between 12 months and 13 months of age^2^; currently, only 89% receive a first dose by age 24 months, and only 84% receive a second dose by age 5 years.^3^ This countrywide statistic conceals marked geographic inequalities linked to deprivation. The WHO recommends that 95% of the population are given two MMR doses to achieve herd immunity and eliminate measles.^4^ The UK lost measles elimination status in 2018 and while this was reinstated in 2021, measles outbreaks in areas with high measles susceptibility in young children in England suggest that this will not be sustained.^5^ Clusters of inequalities in MMR coverage exacerbate existing outbreaks—a large proportion have been in London, an area with both low and profoundly inequitable coverage.^3^

In light of these public health concerns, and with the first dose conferring 93% protection against infection, there has been increasing emphasis on the importance of timely receipt of MMR1.^6^ In the UK, national targets to ensure receipt of first MMR (MMR1) between 12 months and 24 months of age have been recently replaced by a 12–18-month target reflecting this emphasis on timeliness.^7^

It is known that equity in vaccination coverage is impacted by social determinants such as deprivation, ethnicity and area-level variation in healthcare services.^8 9^ There is strong evidence demonstrating that children from more deprived areas are less likely to receive MMR vaccination compared with those living in affluent areas.^10^ We and others^11^ have previously shown that family size is an important determinant of partial or non-immunisation with MMR, suggesting that access to services may play an important role.^12 13^

Identifying factors at a household level can create actionable insights into how services might be tailored to improve receipt of vaccinations.^14^ The current pressures on the UK National Health Service have significantly impacted the delivery of vaccinations in primary care. Therefore, new ways of working to vaccinate the most vulnerable children in a resource-tight setting are needed.^15 16^ We used electronic health records (EHRs) for an ethnically diverse and disadvantaged population, with among the lowest proportion of children receiving MMR1 by 24 months of age in the UK, to investigate whether non-receipt of MMR1 between 12 months and 24 months of age is clustered in households. Specifically, we hypothesised that children with non-receipt of MMR1 between 12 months and 24 months were more likely to share a household with an older child with non-receipt of MMR1 at these ages, independently of the number of children in the household and household composition.

## Methods

### Study design and setting

We conducted a longitudinal observational study using primary care EHRs from 266 general practices in seven North East London (NEL) localities: Barking and Dagenham, City and Hackney, Havering, Newham, Redbridge, Tower Hamlets and Waltham Forest.

### Data sources

Pseudonymised data were provided from the NEL Discovery Data Service (DDS), which receives primary care EHR data in near-real time for all general practices (GPs) in NEL.^17^ Unique property reference numbers (UPRNs) are allocated to all GP-recorded patient addresses in the DDS using a quality-assured and validated address-matching algorithm.^18^ UPRNs are pseudonymised into residential anonymous linking fields (RALF)^19^ using a study-specific encryption key. We used RALFs to link children in households for address records and registrations from 2014 onwards, when data flow for address registrations into NEL DDS commenced. Data were extracted on 23 November 2021.

### Study population

The study population comprised 159 300 children registered with a NEL GP at the time of their second birthday and eligible to receive MMR1 between 1 January 2014 and 28 February 2020. We excluded 17 038 children without a RALF, with a non-residential RALF, with a poor-quality RALF match or with more than one RALF at the time of MMR1 or second birthday, leaving 142 262 children eligible for inclusion (online supplemental figure 1).

### Identifying children sharing a household

We identified older children sharing a household with the 142 262 index children at the index child’s MMR1 date or 24 months of age, whichever is the earliest. Index and older children sharing a RALF at the index child’s MMR1 date or at the index child’s second birthday were considered to share a household. We identified all children in DDS based on the index children’s RALFs and excluded 52 693 children without an older child in the household and 15 516 older children who were already included as index children, leaving 71 509 index children with at least one older child sharing their household at the index child’s MMR1 date or second birthday (online supplemental figure 2). These 71 509 children are henceforth referred to as the ‘linked index cohort’ and the older children with whom they share a household as the ‘linked older children’s cohort’.

The study methodology has been reported against the REporting of studies Conducted using Observational Routinely-collected health Data statement (online supplemental file 2).^20 21^

### Primary outcome

The primary outcome is receipt of MMR1 between 12 months and 24 months of age, which is consistent with the Cover of Vaccination Evaluated Rapidly measures in place during the study period.^22^

We extracted sociodemographic and area-level data for the linked index and linked older child cohorts, together with all clinical events relating to MMR1 procedures (online supplemental table 1). We derived a proxy date of birth from calendar week, month and year of birth by combining the date of the first day of the week of the calendar week of birth with month and year of birth. We excluded duplicated events and events without correct clinical codes. We assumed MMR1 was not given if there was no record of MMR1 being given in the primary care EHR. If a child did not have a record of an MMR1 vaccination, they were linked to a RALF at the time of their second birthday and were defined as children with non-receipt of MMR1.

### Explanatory variable

The main explanatory variable was non-receipt of MMR1 in the linked older child defined as no record of MMR1 given between 12 months and 24 months of age.

### Covariates

#### Individual-level

Individual-level covariates were sex and ethnic group. We categorised the ethnic group of the index children using the National Health Service 5+1 classification using information recorded in the EHR.^23^ We created five mutually exclusive ethnic groups: white (‘white British’, ‘white Irish’ or ‘any other white background’); black (‘black African’, ‘black Caribbean’ or ‘any other black background’); South Asian (‘Indian’, ‘Pakistani’, ‘Bangladeshi’ or ‘Sri Lankan’); mixed/other (‘any other ethnic background’, ‘mixed ethnicity’, ‘Chinese’ or ‘Asian other’); and missing category (ethnicity code in the primary care record missing or ‘not stated’ category selected).

#### Household-level

All household members sharing a household at the index child’s MMR1 date were identified. We excluded households with more than 10 members, only one child or no adults (aged ≥18.0 years). Household information was available for 65 308 households containing index and linked older children.

We categorised household composition using an adapted Harper and Mayhew method^24^ into one of three mutually exclusive categories: working-age adults (aged 18–64 years) with children; single working-age adult with children or at least one working-age and one older adult (aged >65 years) with children (three-generation household). We included households with at least one older adult with children but no working-age adult (skipped generation households) in the three-generation household group.

We calculated the total number of household members, as well as the number of children within a household at the index child’s MMR1 date or 24 months of age for those with no MMR1 date.

#### Area-level

We merged the 2019 Index of Multiple Deprivation (IMD) decile^25^ into the datafile using the 2011 Lower layer Super Output Area, an area with an average population of 1500 people or 650 households, as the linkage field. IMD deciles were concatenated into quintiles from most (1) to least deprived (5).

We compared the linked index cohort (n=71 509) with the cohort of eligible children (n=70 753) not linked to another older child (online supplemental table 2). The linked sample had a lower proportion with receipt of MMR1 between 12 and 24 months of age, were less likely to be from a white ethnic background, from smaller households or from households with two or more working age adults.

### Statistical methods

We calculated the proportion of the linked index and older child cohorts receiving MMR1 between 12 months and 24 months of age. We examined variation in MMR1 receipt in the linked index cohort by individual-level, household-level and area-level characteristics, as well as by MMR1 receipt in the linked older children’s cohort.

We estimated the likelihood of MMR1 vaccination between 12 months and 24 months of age in the linked index cohort using binary logistic regression and estimated OR and 95% CIs for those sharing a household with a linked older child with non-receipt of MMR1 between 12 months and 24 months of age, before and after adjustment for individual-level, household-level and area-level covariates. Covariates with p value <0.1 in the univariable logistic regression models were included in a multivariable logistic regression model following a stepwise model selection strategy. Variables were retained in the final multivariable model if p value *≤*0.05.

We performed three sensitivity analyses. In the first, we changed the definition of the primary outcome to receipt of MMR1 between 12 months and 18 months of age in line with the recently introduced Quality and Outcomes Framework targets, introduced in 2021.^26^ In the second, we excluded households containing index and linked older children with an age gap of more than 5 years. In the third, we extended the age range for MMR1 receipt in the index children from 12–24 months to 11–25 months to allow for potential misclassification of ages related to method for assigning date of birth. We performed post-hoc power calculations to determine an appropriate sample size to power our study for the primary outcome. All analyses were conducted using R Studio.^27^

Post-hoc power calculations demonstrated that a sample size of 52 000 in the index cohort would provide 90% power to detect a two percentage point difference significant at the 1% level in MMR1 receipt between 12 months and 24 months of age in the index child between those with and without a linked older child with no MMR1 receipt between 12 months and 24 months.

### Patient and public involvement

We involved patients and the public in the communication of study results and dissemination within the local community, in line with accepted principles from the UK Standards for Public Involvement.^28^ The aim was to raise awareness of the importance of inequalities in timely childhood vaccinations. We established a patient advisory group, comprising six parents, to coproduce dissemination materials. The patient and public involvement group reflected on vaccination inequalities, the study design and how results were delivered. Participants expressed reservations about the categorisation of ethnic group and whether more granular categories could be used in future research. They discussed communication and visualisation of results. Dissemination of results is ongoing and informed by advice about accessing seldom-heard as well as existing community groups.

## Results

The index cohort comprised 71 509 children (51% boys) of whom 11 658 (16.4%) had not received MMR1 vaccine between 12 months and 24 months of age. Children in the index cohort who did not receive MMR1 between 12 months and 24 months of age were more likely to live with a linked older child who similarly had not received MMR1 between 12 months and 24 months of age (table 1). Index children receiving MMR1 between 12 months and 24 months of age were more likely to be from South Asian ethnic groups or living in households with fewer adults and fewer children, or in households with two or more working age adults or three-generation households. Children in single adult households or in households with a larger number of children were less likely to receive MMR1 between 12 months and 24 months. There was a marked gradient in timely MMR1 receipt by IMD quintile with an absolute difference of 7.3% in MMR1 receipt between 12 months and 24 months of age between the least and most deprived quintiles.

**Table 1:** MMR1 receipt in linked index children by individual-level, household-level and area-level characteristics

	Vaccinated			Non-vaccinated			All linked index children		
	n=59 851 (83.6%)			n=11 658 (16.4%)			n=71 509		
	Received first MMR between 12 months and 24 months of age			Did not receive first MMR between 12 months and 24 months of age					
	*N*	%	95% CI	*N*	%	95% CI	*N*	%	95% CI
MMR1 status of oldest child									
Vaccinated	53 198	88.4	88.1, 88.6	6987	11.6	11.3, 11.9	60 185	84.2	83.9, 84.4
Non-vaccinated	6653	58.8	57.8, 59.7	4671	41.2	40.3, 42.2	11 324	15.8	15.6, 16.1
Individual covariates									
Ethnic background									
South Asian	16 963	88.0	87.6, 88.5	2305	12.0	11.5, 12.4	19 268	25.5	25.1, 25.8
White	16 625	83.8	83.3, 84.3	3219	16.2	15.7, 16.7	19 844	28.3	27.9, 28.6
Black or Black-British	5703	82.2	81.2, 83.1	1238	17.8	16.9, 18.7	6941	10.0	9.8, 10.2
Mixed and other	4847	78.8	77.8, 79.8	1303	21.2	20.8, 22.2	6150	8.5	8.3, 8.7
Missing*	15 713	81.4	80.8, 81.9	3593	18.6	18.1, 19.2	19 306	27.7	27.4, 28.1
Sex									
Female	29 399	84.0	83.6, 84.3	5614	16.0	15.6, 16.4	35 013	48.9	48.5, 49.3
Male	30 452	83.4	83.0, 83.8	6044	16.6	16.2, 16.9	36 496	51.1	50.7, 51.4
Household-level covariates									
Household size									
3–4	18 695	86.1	85.7, 86.6	2976	13.9	13.4, 14.3	21 671	30.3	30.0, 30.6
5–7	26 867	84.0	83.6, 84.4	5097	16.0	15.6, 16.4	31 964	44.8	44.4, 45.2
8–10	9397	80.6	79.9, 81.3	2264	19.4	18.7, 20.1	11 661	16.3	16.0, 16.6
Missing*	4881	78.7	77.7, 79.7	1320	21.3	20.3, 22.3	6201	8.6	8.4, 8.8
Household composition									
Two working age adults with children	42 380	84.6	84.3, 84.9	7713	15.4	15.1, 15.7	50 093	76.7	76.4, 77.0
Single working age adult with children	7699	81.5	80.7, 82.3	1747	18.5	17.7, 19.3	9446	14.5	14.2, 14.7
Three-generational household	4891	84.8	83.8, 85.7	878	15.5	14.5, 16.4	5769	8.8	8.6, 9.0
Missing*	4881	78.7	77.7, 79.7	1320	21.3	20.3, 22.3	6201	8.6	8.4, 8.8
Number of children in household									
2–3	43 968	85.4	85.0, 85.7	7527	14.6	14.3, 14.9	51 495	72	71.7, 72.3
4–6	10 669	80.2	79.5, 80.8	2629	19.8	19.2, 20.5	13 298	18.7	18.4, 19.0
7–9	333	64.7	60.4, 68.8	182	35.3	31.2, 39.6	515	0.7	0.6, 0.8
Missing*	4881	78.7	77.7, 79.7	1320	21.3	20.3, 22.3	6201	8.6	8.4, 8.8
Area level covariates									
Index of Multiple Deprivation Quintile									
1 (most deprived)	23 861	83.9	83.5, 84.3	4587	16.1	15.7, 16.5	28 448	40	39.7, 40.3
2	23 512	82.3	81.7, 82.8	5052	17.7	17.2, 18.1	28 564	39.8	39.5, 40.1
3	7600	83.9	83.2, 84.7	1454	16.1	15.3, 16.8	9054	12.6	12.4, 12.8
4	3345	88.9	87.9, 89.9	417	11.1	10.1, 12.1	3762	5.2	5.0, 5.4
5 (least deprived)	1533	91.2	89.7, 92.5	148	8.8	7.5, 10.2	1681	2.3	2.2, 2.4

* Children that could not be linked to other members of the household apart from the oldest child were documented as having household demographics as ‘Missing’.

MMR1, first measles, mumps and rubella dose.

**Figure 1:**
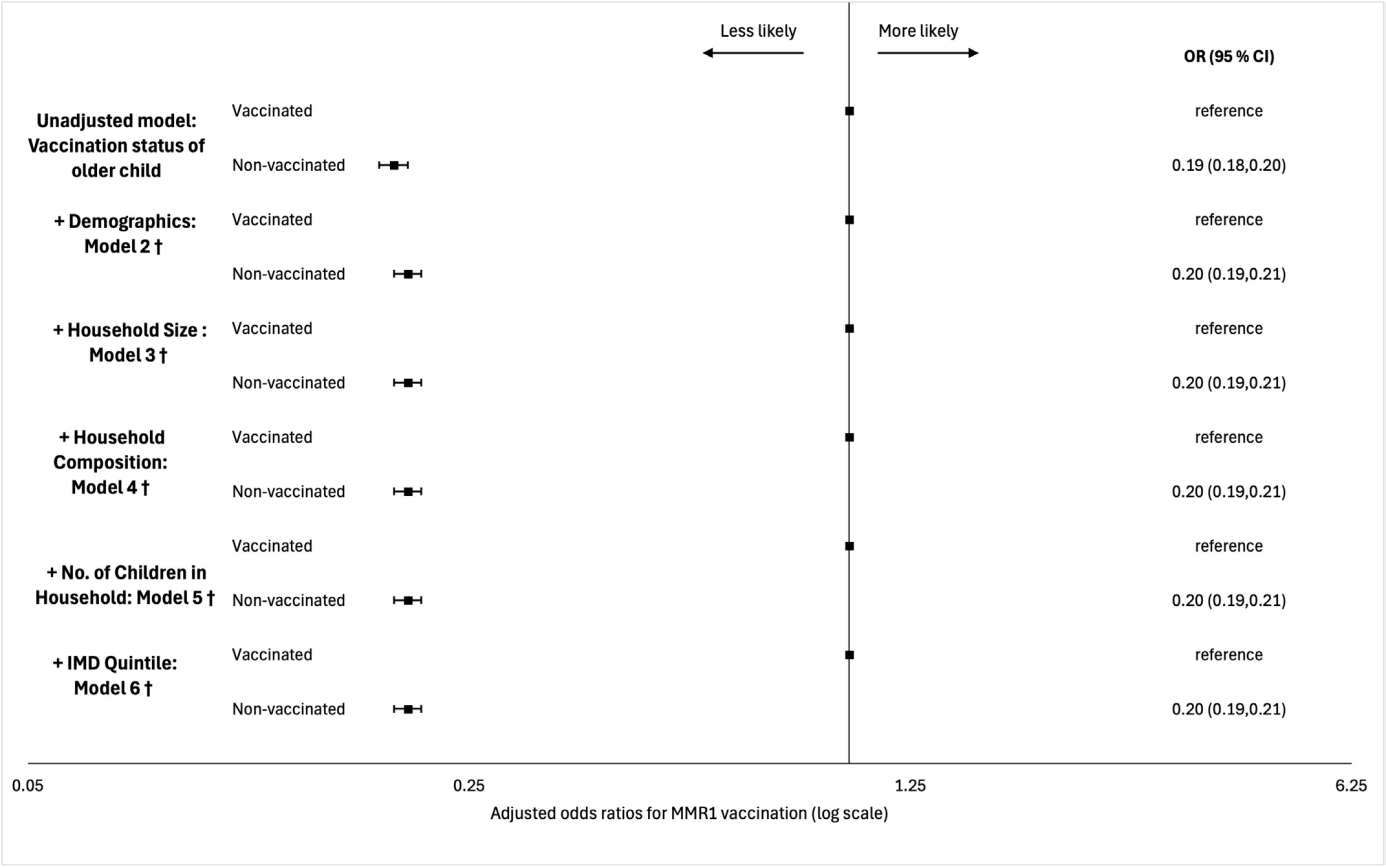
Forest Plot of MMR1 vaccination ORs and 95% CIs between 12 months and 24 months of age using stepwise binary logistic regression. †Model 1: vaccination status of older child sharing household with index child. Model 2: model 1 + sex + ethnicity of index child. Model 3: model 2 + household size. Model 4: model 3 + household composition. Model 5: model 4 + number of children in the household. Model 6: model 5 + Index of Multiple Deprivation quintile. Vaccinated, signifies receipt of MMR1 between 12 months and 24 months of age. MMR1, first measles, mumps and rubella dose.

In the unadjusted model, MMR1 receipt between 12 months and 24 months of age was less likely among children in the linked index cohort sharing a household with a linked older child with no MMR1 receipt between 12 months and 24 months of age (OR: 0.19, 95% CI: 0.18, 0.20). The effect size and direction did not change after stepwise introduction of individual-level, household-level and area-level covariates, resulting in an adjusted OR of 0.20 (0.19,0.21) in the final model (figure 1; online supplemental table 3).

**Figure 2:**
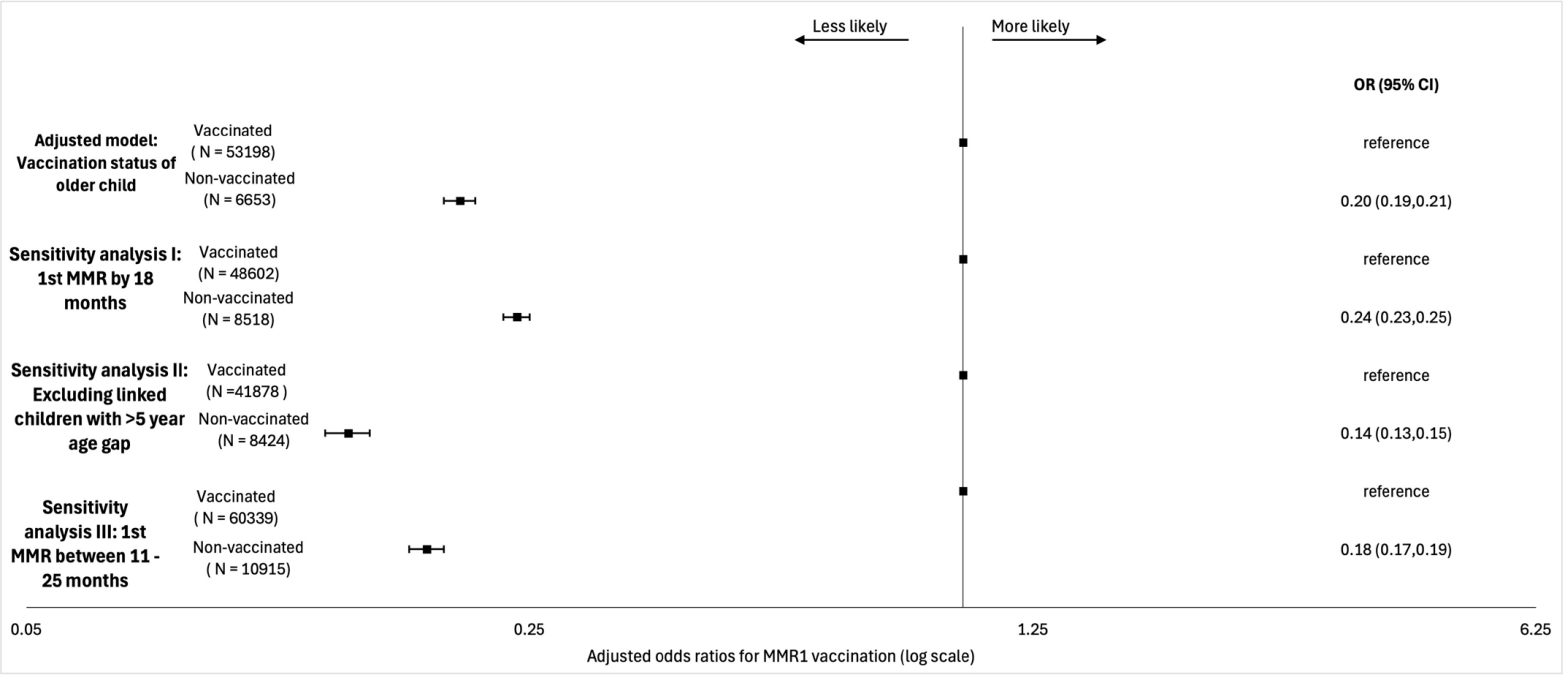
Forest plot of MMR1 vaccination ORs and 95% CIs from main model and from sensitivity analyses. Vaccinated, signifies receipt of MMR1 between 12 months and 24 months of age. MMR1, first measles, mumps and rubella dose.

In sensitivity analyses (figure 2), the proportion of index children with MMR1 receipt between 12 months and 18 months of age (79.2%; 95% CI: 78.9, 79.5) was, as expected, lower than the proportion with MMR1 receipt between 12 months and 24 months (83.6%; 95% CI: 83.3, 83.9) (online supplemental table 4). Associations were weaker in sensitivity analyses using this measure as the primary outcome (OR: 0.24; 0.23, 0.25) (online supplemental table 5). By contrast, associations were stronger in sensitivity analyses restricted to households containing index children and linked older cohort children with an age gap of less than 5 years: OR: 0.14 (0.13,0.15) (online supplemental table 6). Sensitivity analyses extending the age range for MMR1 receipt to 11–25 months did not change the main findings: OR: 0.18 (0.17,0.19) (online supplemental table 7).

While our study focused on MMR1 receipt within the UK recommended age range at the time of the study, it is possible that children were vaccinated before or after the recommended age range. We searched for MMR1 dates for those with no MMR1 date within the 12–24 month age range. Of the 11 658 index children with no MMR1 receipt between 12–24 months, 516 (4.4%) had a MMR1 record before age 12 months, 2893 (24.8%) between 25 months and 40 months of age (equivalent to 3 years and 4 months when children become eligible for the second dose), 749 (6.4%) received MMR1 after 40 months of age and 7500 (64.3%) had no record of MMR1 receipt in the EHR by November 2021 when data were extracted (table 2). This suggests that just over one third of index children did eventually receive MMR1 but significantly later than the recommended age. Almost half (47%) of the linked older children without MMR1 receipt between 12 months and 24 months of age also eventually received MMR1, and this was also significantly later than the recommended age.

**Table 2:** MMR1 receipt in linked index and older children without MMR1 receipt between 12 months and 24 months of age

Non-vaccinated groups	Index child (n=11 658)	%	Older child (n=11 324)	%
MMR1 receipt <12 months of age	516	4.4	993	8.8
MMR1 receipt between 24 months and 40 months of age	2893	24.8	2642	23.3
MMR1 receipt >40 months of age	749	6.4	1689	14.9
No record of MMR1 receipt in period of follow-up	7500	64.3	6000	53.0
Total	11 658	100.0	11 324	100.0

MMR1, first measles, mumps and rubella dose.

## Discussion

We have shown that 16% of children from an English urban, disadvantaged and multiethnic population with low MMR1 coverage do not receive MMR1 between the recommended age interval of 12 months and 24 months, and that they are less likely to do so if they share a household with an older child who did not receive MMR1 between age 12 months and 24 months. This association was independent of ethnic group, number of children in the household, household composition and area-level deprivation and was strengthened when analyses were confined to household children with an age gap of less than 5 years. We also found that children in single adult households or in households with a larger number of children are less likely to receive MMR1 between 12 months and 24 months of age, consistent with findings from previous studies reporting household characteristics of children with delayed or non-MMR1 receipt. These findings suggest that caregivers’ actions related to attendance for child vaccinations may be consistent across children in the household, particularly among children who are close in age.

While we examined MMR1 receipt within the UK recommended age range of 12 months to 24 months in place at the time of our study, we were able to show that one third of index children did receive MMR1 at both younger and older ages. There are a number of explanations for this. UK vaccine guidance states that MMR1 may be given under 12 months of age in the context of outbreaks or exposure to measles. However, as there is evidence that this doesn’t produce a strong antibody response, it is recommended that MMR1 must be given again within the scheduled age range.^2^ Parents may not agree to a second MMR1, especially if this was given close to the first birthday. Furthermore, a proportion of MMR1 events under 12 months of age were assigned an improbable date (eg, given at birth date), and we are aware that GP practices may use this to record vaccines given in other countries for which the caregiver is unable to provide a date. London includes a significant proportion of children who are non-UK born and who migrate after the age of primary immunisations, many of whom anecdotally also spend periods back in their country of birth.^29 30^ This complicates administration and recording of vaccines and may create different expectations among parents or caregivers regarding vaccine schedules. Opportunistic catch-up of MMR1 has also been initiated on a number of occasions, and appointments for the second dose may be the opportunity to give the first dose: almost one quarter of index and linked older children were given MMR1 between 24 months and 40 months of age. So, while we were unable to confirm MMR1 receipt in two-thirds of index and one half of linked older children, a significant proportion were delayed rather than never immunised.

This is to our knowledge the first study to examine associations within households of MMR1 timeliness, so direct comparisons with existing literature are not possible. Previous studies have found that vaccine coverage is lower in families with larger numbers of children and in single-parent households.^31 32^ It has been suggested that the main drivers of vaccination delay in these households are access-based, with vaccination services and appointments less suitable for families with larger numbers of children or for parents requiring more flexible clinic appointments.^12 33^ Vaccination delay may also be non-intentional: parents may delay vaccinations due to a child’s illness.^34^ This may explain some of the factors driving delayed MMR1 receipt in our study.

There may be other reasons for delayed MMR1 receipt. Qualitative research around reasons for delayed, partial or non-vaccination of children highlights the importance for parents of shared decision-making with clinicians and the strong association between trust in healthcare professionals and vaccine hesitancy in parents or caregivers. Parents or caregivers who have some trust in the information given by healthcare professionals may delay rather than completely refuse a child’s vaccination, and this may be a consistent factor for all children in the household.^35^ One study looking at decision-making between adults and adolescents in a household for the MenACWY vaccination found that information gathering outside of a healthcare setting, even prior to invitation for vaccination, significantly impacted the decision made.^36^

Vaccinations can also be delayed by parents if they feel that information around the safety of a vaccine is insufficient, or if they have concerns about overburdening a child’s immune system.^37 38^ Parental or caregiver disagreement around childhood vaccination may also contribute to delay.^13^

Further qualitative research is needed to tease out the likely heterogeneous reasons for MMR1 delay or non-receipt at a household-level and to understand household factors that interact with access and the decision-making process.^39^ Delay in primary vaccinations against diphtheria, pertussis, polio, tetanus and *Haemophilus influenzae* has been shown to be associated with an incomplete vaccination schedule by 24 months of age.^40^ We were not able to examine this in our study.

### Implications for practice

Our study has demonstrated that delay in MMR1 receipt is strongly clustered within households. This lack of timely protection or any protection within households increases the risk of measles outbreaks. This suggests the need for household-based interventions to improve vaccination coverage and timeliness. Knowing the household composition of children with delayed or non-vaccination can allow a healthcare professional (HCP) to tailor their approach to organising vaccination appointments. For example, if it is known that there is more than one child in the household needing vaccination, a HCP can arrange an appropriate appointment for two children at one time. In England, the EHR in GPs allows a HCP to view other patients registered at the same address as the selected patient.

Household-based interventions could also be considered by public health and service commissioners. Setting up services tailored to households with non-vaccinated or partially-vaccinated children aligns with documented interventions recommended to improve vaccination coverage.^41^ The same principle applies to providing wider public health education about vaccination for these households: interventions can be more targeted when non-vaccinated or partially-vaccinated households are identified. Emerging interventions using enhanced information and educational programmes and vaccination delivery by health visitors could be tailored to target more vulnerable households.^42^ Evidence from adolescent/adult decision-making about vaccines in a household reinforces the importance of giving parents relevant information before the offer of vaccination from a healthcare provider.^36^

The existing literature cites multicomponent interventions as the most effective interventions for increasing vaccination coverage in deprived communities with intersectional inequalities, including information, education and recall measures.^39^ Robust recall methods are cited as an effective way to vaccinate children with delayed vaccinations.^43^ We have shown that a quality improvement programme that aims to improve timeliness and equity of preschool immunisations in NEL, focussing on data-enabled call and recall for immunisation, is effective.^44^

### Future research

We have shown that non-receipt of MMR1 between 12 months and 24 months of age is clustered in households. However, a significant proportion of children in our study ultimately received MMR1 in the preschool years and later childhood, with no clear evidence of MMR1 receipt in the remainder. Qualitative research is needed to understand the decision-making processes underlying this heterogeneous group. Similar research in demographically different areas of the UK may help understand the extent to which these findings are generalisable to households in a different socioeconomic context.

### Strengths and limitations

The strengths of our study include the use of a novel method to create households securely while maintaining privacy, as well as having access to a large population with EHRs for a geographically contiguous area. Additionally, we have access to high-quality MMR data that are recorded accurately in the EHR through data recording templates.^45^ The codeset used to identify MMR1 in the EHR was validated. We used robust statistical methods to assess relationships between the exposure and outcome variables, and we selected a time period before lockdowns due to the Coronavirus pandemic disrupted access to healthcare in England (March 2020).

We were not able to examine associations with delayed receipt of primary vaccinations against diphtheria, pertussis, polio, tetanus and *H.influenzae*. More granular categorisation of ethnic groups, as suggested by our patient and public involvement group, was not possible due to limited sample size. Processes of decision-making about vaccines may have differed between the linked index and older children. However, associations between the vaccination status of a younger and linked older child strengthened when restricted to children with an age interval of less than 5 years.

## Conclusion

There is strong concordance in MMR1 vaccine delay or non-receipt between children sharing the same household in a region with the lowest MMR vaccination coverage in the UK.^3^ These findings have implications for the planning and delivery of vaccination services that consider children in their household context.

## Supplementary material

10.1136/bmjopen-2024-097559 online supplemental file 1

## Data Availability

Data may be obtained from a third party and are not publicly available.
