# Supplementary material for: Household determinants of delayed MMR vaccination: longitudinal analysis using electronic health records in North East London, UK
Source: BMJ Open. 2025 May 2;15(5):e097559. doi: 10.1136/bmjopen-2024-097559 (PMC12049873; doi:10.1136/bmjopen-2024-097559)
Supplement: online supplemental file 1 [file bmjopen-15-5-s001.docx]

**Household determinants of delayed MMR vaccination: longitudinal analysis using electronic health records in north east London, United Kingdom**

Milena Marszalek^1^, Nicola Firman^1^, Marta Wilk^1^, Ana Gutierrez^1^, Kelvin Smith^1^, Carol Dezateux^1^

^1^Centre for Primary Care, Wolfson Institute of Population Health, Faculty of Medicine and Dentistry, Queen Mary University of London, Yvonne Carter Building, 58 Turner Street, London, E1 2AB

## **Supplementary file 1 – additional tables and figures**

**Figure S1-Inclusion and exclusion criteria for sample population with a valid Residential Anonymised Linkage Field (RALF)**


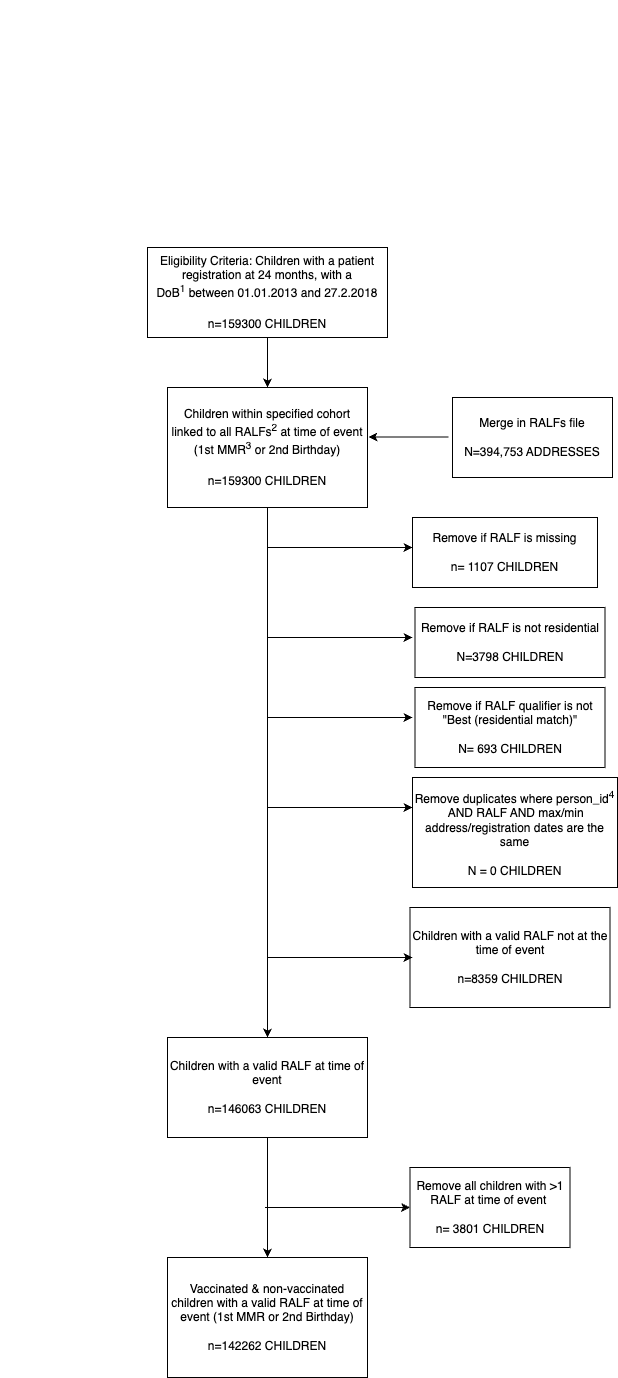


##

1. Date of Birth
2. Residential Anonymised Linkage Field
3. Measles, Mumps & Rubella vaccination
4. Individual person identifier

## **Figure S2-** **Inclusion and exclusion criteria for linking index and older children**
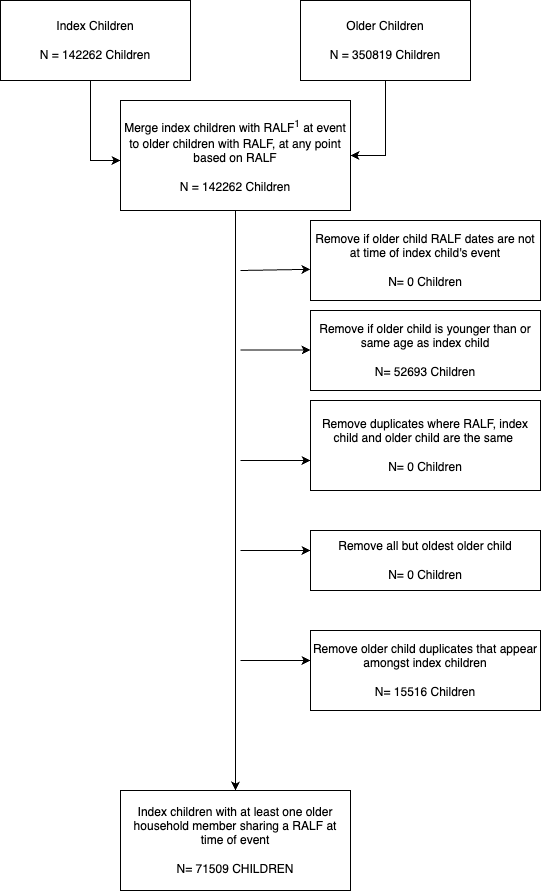


^1^  Residential Anonymised Linkage Field

## **Table S1- Systematized Nomenclature of Medicine (SNOMED) clinical codes for first Measles, Mumps and Rubella vaccination procedures**

Events recorded in the primary care electronic heath record using another clinical coding system (e.g. Read v2 or EMIS local codes) have been mapped to relevant SNOMED codes within the Discovery Data Service. This ensures that searching the database using SNOMED codes captured all events regardless of the clinical coding system used.

| SNOMED concept ID | Other code | Clinical coding scheme | Code description |
| --- | --- | --- | --- |
| 38598009 | 38598009 | SNOMED | Measles-mumps-rubella vaccination (procedure) |
|  | 65M1. | Read v2 | Measles/mumps/rubella vaccn. |
|  | ^ESCT1405772 | EMIS local | Administration of measles and mumps and rubella vaccine |
| 47435007 | 47435007 | SNOMED | Measles vaccination (procedure) |
|  | 65A.. | Read v2 | Measles vaccination |
|  | 65A1. | Read v2 | Measles vaccination |
|  | ZV042 | Read v2 | [V]Measles vaccination |
|  | ^ESCT1405845 | EMIS local | Administration of measles vaccine |
| 50583002 | 50583002 | SNOMED | Mumps vaccination (procedure) |
|  | 65F5. | Read v2 | Mumps vaccination |
|  | ZV046 | Read v2 | [V]Mumps vaccination |
|  | ^ESCT1405876 | EMIS local | Administration of mumps vaccine |
| 82314000 | 65B.. | Read v2 | Rubella vaccination |
|  | ZV043 | Read v2 | [V]Rubella vaccination |
|  | ^ESCT1406118 | EMIS local | Administration of rubella vaccine |
| 170364006 | 65A2. | Read v2 | Measles vaccin.+immunoglobulin |
| 432636005 | ^ESCT1408534 | EMIS local | Administration of measles and mumps and rubella and varicella virus vaccine |
| 871909005 | ^ESCT1397548 | EMIS local | Administration of first dose of measles and mumps and rubella and varicella virus vaccine |
| 150971000119104 | ZV064 | Read v2 | [V]Measles-mumps-rubella (MMR) vaccination |
| 308081000000105 | 65M10 | Read v2 | First MMR (measles mumps and rubella) vaccination |
|  | Xaeec | Read v3 | First MMR (measles mumps and rubella) vaccination |
|  | ^ESCTME809974 | EMIS local | Measles mumps and rubella vaccination - first dose |
| 505001000000109 | 9ki1. | Read v2 | MMR catch-up vaccination - enhanced services administration |
|  | XaQPr | Read v3 | Measles mumps rubella catch-up vaccination |
| 571591000119106 | ^ESCT1409651 | EMIS local | Administration of live attenuated measles mumps and rubella vaccine |
| 1037251000000100 | 65M11 | Read v2 | First MMR vaccination given by other healthcare provider |
|  | Xaeeq | Read v3 | First MMR vaccination given by other healthcare provider |

We included clinical codes relating to administration of mono-components of the first MMR vaccination. After removal of duplicate data entries and merging to the study cohort, 584989 children had a clinical code for measles vaccination, and two for mumps vaccination, as opposed to a combined MMR vaccination.

## **Table S2- Characteristics of linked and unlinked cohorts by individual-, household- and area level variables**

|  | Linked cohort (n = 71509) | | | Unlinked cohort (n = 70753 ) | | |
| --- | --- | --- | --- | --- | --- | --- |
|  | N | % | 95% CI^1^ | N | % | 95% CI^1^ |
| MMR1^2^ status of oldest child | | | | | | |
| Vaccinated | 59851 | 83.6 | 83.3,83.9 | 60512 | 85.5 | 85.3,85.8 |
| Non-vaccinated | 11658 | 16.4 | 16.1,16.6 | 10240 | 14.5 | 14.2,14.7 |
| Individual covariates | | | | | | |
| Ethic Background | | | | | | |
| South Asian | 19268 | 25.5 | 25.1,25.8 | 16073 | 22.7 | 22.4,23 |
| White | 19844 | 28.3 | 27.9,28.6 | 23536 | 33.3 | 32.9,33.6 |
| Black or Black British | 6941 | 10.0 | 9.8,10.2 | 5467 | 7.7 | 7.5,7.9 |
| Mixed and Other | 6150 | 8.5 | 8.3,8.7 | 6869 | 9.7 | 9.5,9.9 |
| Missing** | 19306 | 27.7 | 27.4,28.1 | 18807 | 26.6 | 26.3,26.9 |
| Sex | | | | | | |
| Female | 35013 | 48.9 | 48.5,49.3 | 34885 | 49.3 | 48.9,49.7 |
| Male | 36496 | 51.1 | 50.7,51.4 | 35867 | 50.7 | 50.3,51.1 |
| Household-level covariates | | | | | | |
| Household size | | | | | | |
| 3 to 4 | 21683 | 30.3 | 30.0 ,30.6 | 37417 | 52.9 | 52.5,53.3 |
| 5 to 7 | 31964 | 44.7 | 44.3,45.1 | 18976 | 26.8 | 26.5,27.1 |
| 8 to 10 | 11661 | 16.3 | 16,16.6 | 7514 | 10.6 | 10.4,10.8 |
| Missing** | 6201 | 8.7 | 8.5,8.9 | 6845 | 9.7 | 9.4,10.0 |
| Household composition | | | | | | |
| Two working age adults with children | 50093 | 70.0 | 69.7,70.3 | 46906 | 66.3 | 66,66.6 |
| Single working age adult with children | 9446 | 13.2 | 13,13.4 | 10356 | 14.6 | 14.4,14.9 |
| Three-generational household | 5769 | 8.1 | 7.9,8.3 | 6645 | 9.4 | 9.2,9.6 |
| Missing** | 6201 | 8.7 | 8.5,8.9 | 6845 | 9.7 | 9.5,9.9 |
| Number of children in household | | | | | | |
| 2 to 3 | 51495 | 72.0 | 71.7,72.3 | 59151 | 83.6 | 83.3,83.9 |
| 4 to 6 | 13298 | 18.7 | 18.4,19 | 4486 | 6.3 | 6.1,6.5 |
| 7 to 9 | 515 | 0.7 | 0.6,0.8 | 270 | 0.4 | 0.3,0.5 |
| Missing | 6201 | 8.6 | 8.4,8.8 | 6845 | 9.7 | 9.4,10.0 |
| Area level covariates | | | | | | |
| Index of Multiple Deprivation (IMD) quintile | | | | | | |
| IMD 1 (Most deprived) | 28448 | 40.0 | 39.7,40.3 | 26062 | 36.8 | 36.5,37.2 |
| IMD 2 | 28564 | 39.8 | 39.5,40.1 | 28972 | 40.9 | 40.5,41.3 |
| IMD 3 | 9054 | 12.6 | 12.4,12.8 | 9602 | 13.6 | 13.3,13.8 |
| IMD 4 | 3762 | 5.2 | 5.0,5.4 | 4311 | 6.1 | 5.9,6.3 |
| IMD 5 (Least deprived) | 1681 | 2.3 | 2.2,2.4 | 1805 | 2.5 | 2.4,2.6 |

** Children that could not be linked to other members of the household apart from the oldest child were documented as having household demographics as ‘Missing’

^1^CI – Confidence interval

^2^ Vaccinated signifies receipt of MMR1 between 12 and 24 months of age

Table S3- Unadjusted and adjusted odds ratios for 1^st^ Measles, Mumps and Rubella (MMR) vaccination between 12 and 24 months of age, by individual-, household-, and area level characteristics:

|  | **OR***^1^* | **95% CI ^2^** | **p-value** | **OR^1^** | **95% CI ^2^** | **p-value** |  |
| --- | --- | --- | --- | --- | --- | --- | --- |
|  | Unadjusted | | | Adjusted | | |  |
| **MMR1^3^ status of oldest child** | | | | | | | |
| Vaccinated | Reference |  |  | Reference |  |  |  |
| Non-vaccinated | 0.19 | 0.18, 0.20 | <0.001 | 0.20 | 0.19, 0.21 | <0.001 |  |
| **Individual covariates** | | | | | | | |
| **Ethnic background** | | | | | | | |
| South Asian | 1.34 | 1.26, 1.42 | <0.001 | 1.46 | 1.37, 1.55 | <0.001 |  |
| White | Reference |  |  | Reference |  |  |  |
| Black or Black British | 0.88 | 0.82, 0.95 | <0.001 | 0.97 | 0.89, 1.04 | 0.40 |  |
| Mixed and Other | 0.76 | 0.71, 0.82 | <0.001 | 0.83 | 0.77, 0.90 | <0.001 |  |
| Missing | 0.84 | 0.79, 0.88 | <0.001 | 0.87 | 0.82, 0.92 | <0.001 |  |
| **Sex** | | | | | | | |
| Male | Reference |  |  | Reference |  |  |  |
| Female | 0.96 | 0.92, 1.00 | 0.061 | 0.96 | 0.92,1.00 | 0.06 |  |
| **Household level covariates** | | | | | | | |
| **Household size** | | | | | | | |
| 3 to 4 | Reference |  |  | Reference |  |  |  |
| 5 to 7 | 0.88 | 0.84, 0.93 | <0.001 | 0.81 | 0.76, 0.86 | <0.001 |  |
| 8 to 10 | 0.74 | 0.69, 0.79 | <0.001 | 0.71 | 0.66, 0.77 | <0.001 |  |
| Missing** | 0.68 | 0.63, 0.73 | <0.001 | NA | NA | NA |  |
| **Household composition** | | | | | | | |
| Two working age adults with children | Reference |  |  | Reference |  |  |  |
| Single working age adult with children | 0.80 | 0.75, 0.85 | <0.001 | 0.72 | 0.67, 0.77 | <0.001 |  |
| Three generational household | 0.97 | 0.90,1.05 | 0.40 | 0.98 | 0.91,1.07 | 0.70 |  |
| Missing** | 0.74 | 0.69, 0.79 | <0.001 | 0.56 | 0.52, 0.61 | <0.001 |  |
| **Number of children in household** | | | | | | | |
| 2 to 3 | Reference |  |  | Reference |  |  |  |
| 4 to 6 | 0.73 | 0.69, 0.77 | <0.001 | 0.82 | 0.77, 0.87 | <0.001 |  |
| 7 to 9 | 0.42 | 0.35, 0.52 | <0.001 | 0.57 | 0.46, 0.70 | <0.001 |  |
| Missing | 0.71 | 0.66, 0.76 | <0.001 | NA | NA | NA |  |
| Area level | | | | | | | |
| **Index of Multiple Deprivation (IMD) quintile** | | | | | | | |
| IMD1 (Most deprived) | Reference |  |  | Reference |  |  |  |
| IMD2 | 0.93 | 0.89, 0.97 | <0.001 | 0.91 | 0.87, 0.95 | <0.001 |  |
| IMD3 | 1.01 | 0.95, 1.08 | 0.80 | 0.96 | 0.90,1.03 | 0.20 |  |
| IMD4 | 1.40 | 1.25, 1.56 | <0.001 | 1.33 | 1.19,1.48 | <0.001 |  |
| IMD5 (Least deprived) | 1.81 | 1.52, 2.16 | <0.001 | 1.69 | 1.42, 2.02 | <0.001 |  |
| ^1^OR = Odds Ratio, **^2^**CI = Confidence Interval  ^3^ Vaccinated signifies receipt of MMR1 between 12 and 24 months of age | | | | | | | |

** Children that could not be linked to other members of the household apart from the oldest child were documented as having household demographics as ‘Missing’

**Table S4- Sensitivity analysis I: timely Measles, Mumps and Rubella (MMR) vaccination status between 12 and 18 months of age, by individual-, household-, and area level characteristics**

|  | **Vaccinated** | | | **Non-vaccinated** | | | **All Index cohort** | | |
| --- | --- | --- | --- | --- | --- | --- | --- | --- | --- |
|  | **N=56641 (79.2%)** | | | **N=14889 (20.8%)** | | | **N=71530** | | |
|  | *Received first MMR^1^ between 12 and 18 months of age* | | | *Did not receive first MMR between 12 and 18 months of age* | | |  | | |
|  | n | % | 95% CI^2^ | n | % | 95% CI | n | % | 95% CI |
| **MMR1^1^ status of oldest child** | | | | | | | | | |
| Vaccinated | 48602 | 85.8 | 85.5,86.1 | 8039 | 14.2 | 13.9,14.5 | 56641 | 79.2 | 78.9,79.5 |
| Non-vaccinated | 8518 | 57.2 | 56.4,58.0 | 6371 | 42.8 | 42.0,43.6 | 14889 | 20.8 | 20.5,21.1 |
| **Individual covariates** | | | | | | | | | |
| **Ethnic Background** | | | | | | | | | |
| South Asian | 16214 | 84.3 | 83.8,84.9 | 3007 | 15.6 | 15.1,16.2 | 19221 | 26.9 | 26.5,27.2 |
| White | 15834 | 79.9 | 79.3,80.5 | 3978 | 20.1 | 19.5,20.6 | 19812 | 27.7 | 27.4,28.0 |
| Black or Black British | 5342 | 76.9 | 75.9,77.9 | 1605 | 23.1 | 22.1,24.1 | 6947 | 9.7 | 9.5,9.9 |
| Mixed and Other | 4448 | 71.8 | 70.7,72.9 | 1745 | 28.2 | 27.1,29.3 | 6193 | 8.7 | 8.5,8.9 |
| Missing | 14803 | 76.5 | 75.9,77.1 | 4554 | 23.5 | 22.9,24.1 | 19357 | 27.1 | 26.7,27.4 |
| **Sex** | | | | | | | | | |
| Female | 27814 | 79.4 | 79.0,79.8 | 7206 | 20.6 | 20.2,21.0 | 35020 | 49.0 | 48.6,49.3 |
| Male | 28827 | 79.0 | 78.5,79.4 | 7683 | 21.0 | 20.6,21.5 | 36510 | 51.0 | 50.6,51.4 |
| **Household-level covariates** | | | | | | | | | |
| **Household size** | | | | | | | | | |
| 3 to 4 | 17848 | 82.4 | 81.9,82.9 | 3819 | 17.6 | 17.1,18.1 | 21655 | 30.3 | 29.9,30.6 |
| 5 to 7 | 25460 | 79.7 | 79.2,80.1 | 6492 | 20.3 | 19.9,20.8 | 31952 | 44.7 | 44.3,45 |
| 8 to 10 | 8806 | 75.5 | 74.7,76.3 | 2849 | 24.4 | 23.7,25.2 | 11655 | 16.3 | 16,16.6 |
| Missing** | 4527 | 72.4 | 71.2,73.5 | 1729 | 27.6 | 26.5,28.8 | 6256 | 8.7 | 8.5,8.9 |
| **Household composition** | | | | | | | | | |
| Two working age adults with children | 40292 | 80.5 | 80.1,80.8 | 9773 | 19.5 | 19.3,19.9 | 50065 | 70.0 | 69.6,70.4 |
| Single working age adult with children | 7187 | 76.1 | 75.2,77.0 | 2256 | 23.9 | 23.0,24.8 | 9443 | 13.2 | 13,13.4 |
| Three-generational household | 4625 | 80.3 | 79.5,81.6 | 1131 | 19.7 | 18.7,20.8 | 5766 | 8.1 | 7.9,8.3 |
| Missing** | 4527 | 72.4 | 71.2,73.5 | 1729 | 27.6 | 26.5,28.8 | 6256 | 8.7 | 8.5,8.9 |
| **Number of Children in household** | | | | | | | | | |
| 2 to 3 | 41973 | 81.6 | 81.2,81.9 | 9494 | 18.4 | 18.1,18.8 | 51467 | 71.9 | 71.6,72.2 |
| 4 to 6 | 9875 | 74.2 | 73.5,75.0 | 3422 | 25.7 | 25.0,26.5 | 13297 | 18.6 | 18.3,18.9 |
| 7 to 9 | 266 | 52.1 | 47.7,56.5 | 244 | 47.8 | 43.4,52.3 | 510 | 0.7 | 0.6,0.8 |
| Missing** | 4527 | 72.4 | 71.2,73.5 | 1729 | 27.6 | 26.5,28.8 | 6256 | 8.7 | 8.5,8.9 |
| **Area level covariates** | | | | | | | | | |
| **Index of Multiple Deprivation (IMD) Quintile** | | | | | | | | | |
| IMD 1 (Most deprived) | 22451 | 78.9 | 78.4,79.4 | 5998 | 21.0 | 20.6,21.6 | 28449 | 39.8 | 39.4,40.1 |
| IMD 2 | 22180 | 77.6 | 77.1,78.1 | 6390 | 22.4 | 21.9,22.9 | 28570 | 39.9 | 39.6,40.3 |
| IMD 3 | 7273 | 80.3 | 79.4,81.1 | 1786 | 19.7 | 18.9,20.5 | 9059 | 12.7 | 12.4,12.9 |
| IMD 4 | 3238 | 85.9 | 84.8,87 | 530 | 14.0 | 13,15.2 | 3768 | 5.3 | 5.1,5.4 |
| IMD 5 (Least deprived) | 1499 | 89.0 | 87.4,90.5 | 185 | 11.0 | 9.5,12.5 | 1684 | 2.3 | 2.3,2.4 |

^1^ Vaccinated signifies receipt of MMR1 between 12 and 24 months of age

^2^CI- Confidence interval

** Children that could not be linked to other members of the household apart from the oldest child were documented as having household demographics as ‘Missing’

## **Table S5- Sensitivity analysis I- unadjusted and adjusted odds ratios for 1^st^ Measles, Mumps and Rubella vaccination receipt between 12 and 18 months of age**

|  | **OR***^1^* | **95% CI***^2^* | **p-value** | **OR***^1^* | **95% CI***^1^* | **p-value** |
| --- | --- | --- | --- | --- | --- | --- |
|  | Unadjusted | | | Adjusted | | |
| **MMR1^3^ status of oldest child** | | | | | | |
| Vaccinated | Reference |  |  | Reference |  |  |
| Non-vaccinated | 0.22 | 0.21,0.23 | <0.001 | 0.24 | 0.23,0.25 | <0.001 |
| **Individual covariates** | | | | | | |
| **Ethnic Background** | | | | | | |
| South Asian | 1.29 | 1.22,1.36 | <0.001 | 1.41 | 1.34,1.49 | <0.001 |
| White | Reference |  |  | Reference |  |  |
| Black or Black British | 0.83 | 0.78,0.89 | <0.001 | 0.76 | 0.71,0.81 | <0.001 |
| Mixed and Other | 0.69 | 0.64,0.74 | <0.001 | 0.85 | 0.81,0.89 | <0.001 |
| Missing | 0.82 | 0.78,0.86 | <0.001 | 0.92 | 0.86,0.99 | 0.027 |
| **Sex** | | | | | | |
| Female | Reference |  |  | Reference |  |  |
| Male | 0.97 | 0.94,1.01 | 0.20 | 0.97 | 0.94,1.01 | 0.20 |
| **Household level covariates** | | | | | | |
| **Household size** | | | | | | |
| 3 to 4 | Reference |  |  | Reference |  |  |
| 5 to 7 | 0.90 | 0.86,0.94 | <0.001 | 0.83 | 0.79,0.88 | <0.001 |
| 8 to 10 | 0.75 | 0.71,0.79 | <0.001 | 0.75 | 0.70,0.81 | <0.001 |
| Missing** | 0.65 | 0.60,0.69 | <0.001 | NA | NA | NA |
| **Household composition** | | | | | | |
| Two adults with children | Reference |  |  | Reference |  |  |
| Single adult with children | 0.78 | 0.74,0.82 | <0.001 | 0.71 | 0.67,0.76 | <0.001 |
| Three generational household | 0.96 | 0.90,1.04 | 0.30 | 0.97 | 0.90,1.04 | 0.40 |
| Missing** | 0.69 | 0.65,0.74 | <0.001 | 0.53 | 0.49,0.57 | <0.001 |
| **Number of children in household** | | | | | | |
| 2 to 3 | Reference |  |  | Reference |  |  |
| 4 to 6 | 0.71 | 0.68,0.74 | <0.001 | 0.79 | 0.74,0.83 | <0.001 |
| 7 to 9 | 0.35 | 0.29,0.42 | <0.001 | 0.46 | 0.38,0.56 | <0.001 |
| Missing** | 0.66 | 0.62,0.70 | <0.001 | NA | NA | NA |
| **Area level covariates** | | | | | | |
| **Index of Multiple Deprivation (IMD) quintile** | | | | | | |
| IMD 1 (Most deprived) | Reference |  |  | Reference |  |  |
| IMD 2 | 0.95 | 0.91,0.99 | 0.012 | 0.93 | 0.89,0.97 | <0.001 |
| IMD 3 | 1.06 | 1.00,1.13 | 0.050 | 1.00 | 0.94,1.07 | 0.90 |
| IMD 4 | 1.46 | 1.32,1.61 | <0.001 | 1.37 | 1.24,1.52 | <0.001 |
| IMD 5 (Least deprived) | 1.95 | 1.67,2.30 | <0.001 | 1.81 | 1.54,2.13 | <0.001 |
| *^1^* OR = Odds Ratio, ^2^CI = Confidence Interval  ^3^ Vaccinated signifies receipt of MMR1 between 12 and 18 months of age | | | | | | |

** Children that could not be linked to other members of the household apart from the oldest child were documented as having household demographics as ‘Missing’

**Table S6-Sensitivity analyses II- Unadjusted and adjusted odds ratios for 1^st^ Measles, Mumps and Rubella vaccination receipt between 12 and 24 months of age: excluding linked index and older cohort children with an age gap greater than five years**

|  | **OR***^1^* | **95% CI***^2^* | **p-value** | **OR***^1^* | **95% CI***^2^* | **p-value** |
| --- | --- | --- | --- | --- | --- | --- |
|  | Unadjusted | | | Adjusted | | |
| **MMR1^3^ status of oldest child** | | | | | | |
| Vaccinated | Reference |  |  | Reference |  |  |
| Non-vaccinated | 0.13 | 0.12, 0.14 | <0.001 | 0.14 | 0.13,0.15 | <0.001 |
| **Individual covariates** | | | | | | |
| **Ethnic background** | | | | | | |
| South Asian | 1.27 | 1.18, 1.36 | <0.001 | 1.41 | 1.31,1.52 | <0.001 |
| White | Reference |  |  | Reference |  |  |
| Black or Black British | 0.87 | 0.79, 0.95 | 0.003 | 0.98 | 0.90,1.08 | 0.70 |
| Mixed and Other | 0.77 | 0.70, 0.97 | <0.001 | 0.85 | 0.77,0.93 | <0.001 |
| Missing | 0.82 | 0.76, 0.87 | <0.001 | 0.86 | 0.80,0.92 | <0.001 |
| **Sex** | | | | | | |
| Male | Reference |  |  | Reference |  |  |
| Female | 0.97 | 0.92, 1.02 | 0.20 | 0.97 | 0.92,1.02 | 0.20 |
| **Household level covariates** | | | | | | |
| **Household size** | | | | | | |
| 3 to 4 | Reference |  |  | Reference |  |  |
| 5 to 7 | 0.83 | 0.78,0.88 | <0.001 | 0.78 | 0.73,0.84 | <0.001 |
| 8 to 10 | 0.71 | 0.66,0.77 | <0.001 | 0.71 | 0.64,0.79 | <0.001 |
| Missing** | 0.68 | 0.62,0.74 | <0.001 | NA | NA | NA |
| **Household composition** | | | | | | |
| Two working age adults with children | Reference |  |  | Reference |  |  |
| Single working age adult with children | 0.80 | 0.74, 0.86 | <0.001 | 0.71 | 0.65,0.77 | <0.001 |
| Three-generational household | 0.98 | 0.89, 1.07 | 0.60 | 0.99 | 0.89,1.09 | 0.80 |
| Missing** | 0.77 | 0.72, 0.84 | <0.001 | 0.57 | 0.52,0.63 | <0.001 |
| **Number of children in household** | | | | | | |
| 2 to 3 | Reference |  |  | Reference |  |  |
| 4 to 6 | 0.70 | 0.65, 0.74 | <0.001 | 0.80 | 0.74, 0.86 | <0.001 |
| 7 to 9 | 0.49 | 0.40, 0.60 | <0.001 | 0.65 | 0.52, 0.81 | <0.001 |
| Missing** | 0.72 | 0.67, 0.78 | <0.001 | NA | NA | NA |
| **Area level covariates** | | | | | | |
| **Index of Multiple Deprivation (IMD) quintile** | | | | | | |
| IMD 1 (Most deprived) | Reference |  |  | Reference |  |  |
| IMD 2 | 0.96 | 0.91, 1.02 | 0.20 | 0.93 | 0.88,0.99 | 0.018 |
| IMD 3 | 1.09 | 1.01, 1.19 | 0.029 | 1.03 | 0.95,1.12 | 0.50 |
| IMD  4 | 1.54 | 1.35, 1.75 | <0.001 | 1.43 | 1.26,1.64 | <0.001 |
| IMD 5 (Least deprived) | 1.98 | 1.63, 2.44 | <0.001 | 1.82 | 1.49,2.24 | <0.001 |
| *^1^* OR = Odds Ratio, ^2^CI = Confidence Interval  ^3^ Vaccinated signifies receipt of MMR1 between 12 and 24 months of age | | | | | | |

##

** Children that could not be linked to other members of the household apart from the oldest child were documented as having household demographics as ‘Missing’

## **Table S7-Sensitivity analyses III- Unadjusted and adjusted odds ratios in multivariable analysis: 1^st^ Measles, Mumps and Rubella vaccination receipt between 11–25 months of age**

|  | **OR***^1^* | **95% CI***^2^* | **p-value** | **OR***^1^* | **95% CI***^2^* | **p-value** |
| --- | --- | --- | --- | --- | --- | --- |
|  | Unadjusted | | | Adjusted | | |
| **MMR1^3^ status of oldest child** | | | | | | |
| Vaccinated | Reference |  |  | Reference |  |  |
| Non-vaccinated | 0.16 | 0.16, 0.17 | <0.001 | 0.18 | 0.17, 0.19 | <0.001 |
| **Individual covariates** | | | | | | |
| **Ethnic background** | | | | | | |
| Asian or Asian British | 1.33 | 1.25, 1.42 | <0.001 | 1.46 | 1.37, 1.55 | <0.001 |
| White | Reference |  |  | Reference |  |  |
| Black or Black British | 0.88 | 0.82, 0.96 | 0.002 | 0.96 | 0.89, 1.04 | 0.40 |
| Mixed and Other | 0.76 | 0.71, 0.82 | <0.001 | 0.83 | 0.77, 0.90 | <0.001 |
| Missing | 0.84 | 0.80, 0.89 | <0.001 | 0.88 | 0.83, 0.93 | <0.001 |
| **Sex** | | | | | | |
| Male | Reference |  |  | Reference |  |  |
| Female | 0.96 | 0.92, 1.01 | 0.084 | 0.96 | 0.92, 1.01 | 0.085 |
| **Household level covariates** | | | | | | |
| **Household size** | | | | | | |
| 3 to 4 | Reference |  |  | Reference |  |  |
| 5 to 7 | 0.90 | 0.85, 0.94 | <0.001 | 0.81 | 0.76, 0.87 | <0.001 |
| 8 to 10 | 0.74 | 0.69, 0.79 | <0.001 | 0.71 | 0.65, 0.77 | <0.001 |
| Missing** | 0.62 | 0.57, 0.67 | <0.001 | N/A | N/A | N/A |
| **Household composition** | | | | | | |
| Two working age adults with children | Reference |  |  | Reference |  |  |
| Single working age adult with children | 0.79 | 0.74, 0.84 | <0.001 | 0.71 | 0.66, 0.76 | <0.001 |
| Three-generational household | 0.96 | 0.89, 1.05 | 0.40 | 0.98 | 0.90, 1.07 | 0.60 |
| Missing** | 0.67 | 0.62, 0.71 | <0.001 | 0.51 | 0.47,0.55 | <0.001 |
| **Number of Children in household** | | | | | | |
| 2 to 3 | Reference |  |  | Reference |  |  |
| 4 to 6 | 0.74 | 0.70, 0.78 | <0.001 | 0.83 | 0.78, 0.88 | <0.001 |
| 7 to 9 | 0.42 | 0.34, 0.52 | <0.001 | 0.57 | 0.46, 0.71 | <0.001 |
| Missing** | 0.64 | 0.60, 0.69 | <0.001 | NA | NA | NA |
| **Area level covariates** | | | | | | |
| **Index of Multiple Deprivation (IMD) quintile** | | | | | | |
| IMD 1 (Most deprived) | Reference |  |  | Reference |  |  |
| IMD 2 | 0.92 | 0.88, 0.97 | 0.001 | 0.91 | 0.87, 0.95 | <0.001 |
| IMD 3 | 0.99 | 0.92, 1.06 | 0.70 | 0.94 | 0.88, 1.01 | 0.076 |
| IMD 4 | 1.34 | 1.20, 1.50 | <0.001 | 1.27 | 1.14, 1.43 | <0.001 |
| IMD 5 (Least deprived) | 1.85 | 1.54,2.23 | <0.001 | 1.73 | 1.44, 2.09 | <0.001 |
| *^1^* OR = Odds Ratio, ^2^CI = Confidence Interval  ^3^ Vaccinated signifies receipt of MMR1 between 11 and 25 months of age | | | | | | |

** Children that could not be linked to other members of the household apart from the oldest child were documented as having household demographics as ‘Missing’
