# Supplementary material for: Household determinants of delayed MMR vaccination: longitudinal analysis using electronic health records in North East London, UK
Source: BMJ Open. 2025 May 2;15(5):e097559. doi: 10.1136/bmjopen-2024-097559 (PMC12049873; doi:10.1136/bmjopen-2024-097559)
Supplement: Supplementary file 2 [file bmjopen-2024-097559.reviewer_comments.pdf]

PEER REVIEW HISTORY

BMJ Open publishes all reviews undertaken for accepted manuscripts. Reviewers are asked to complete a checklist review form and are provided with free text boxes to elaborate on their assessment. These free text comments are reproduced below.

ARTICLE DETAILS

Title (Provisional)

Household determinants of delayed MMR vaccination: longitudinal analysis using electronic health records in north east London, United Kingdom

Authors

Marszalek, Milena; Firman, Nicola; Wilk, Marta; Gutierrez, Ana; Smith, Kelvin; Dezateux, Carol

---

VERSION 1 - REVIEW

**Reviewer**	**1**
**Name**	**Chen, Xinlei**
**Affiliation**	**University of Pittsburgh, Department of Biostatistics**
**Date**	**23-Dec-2024**
**COI**	**None**

---

1. The author used Poisson regression to examine the association between MMR1 receipt between ages 12 and 24 months and non-receipt of MMR1 between 12 and 24 months in the oldest child sharing the household. However, Poisson regression requires the assumption of mean and variance equality. Is this assumption verified? I would also want to see model diagnoses included.

2. The author stated that "The proportion of index children with MMR1 receipt by age 18 months was, as expected, lower than the proportion with MMR1 receipt by age 24 months: 79.2%, 95% CI: 78.9,79.5". I suggest the author report the result using formal language, not connecting all the statistics with a colon at the end of the sentence.

3. The author stated that "While our study focussed on MMR1 receipt within the UK recommended age range at the time of the study, it is possible that children were vaccinated at older ages". Given the context, I believe the author meant to say "it is possible that children were vaccinated at younger or older ages".

**Reviewer**	**2**
**Name**	**Liu, Lianhua**

Affiliation **Hainan Medical University School of Public Health, Department of Biostatistics**

Date **25-Dec-2024**

COI **None**

Thanks so much for your article. The primary objective of this study is to examine whether delay in first MMR (MMR1) receipt is associated with sharing a household with an older child with delayed MMR1 receipt. The research design idea is reasonable overall, but there are some details that need to be further modified.

1. In the study population you included(71,509), the proportion of non-vaccinated individuals is 15.9% (11,658), which is not too rare. Why didn't you consider using logistic regression? What are the advantages of Poisson regression in this situation?
2. The Poisson regression model requires that the mean equals the variance of the dependent variable. Does the data exhibit overdispersion?
3. I didn't see the goodness-of-fit metrics for the Poisson regression. It would be better to append the results of univariate Poisson regressions and multivariate Poisson regression as supplementary materials to the end of the paper.
4. Please clarify whether the study population is "eligible to receive MMR1 between 1st January 2020 and 28th February 2020" or "eligible to receive MMR1 between 1st January 2014 and 28th February 2020".
5. In the abstract's methods section, you mentioned using "Poisson regression to calculate MMR1 prevalence ratios (PR) and 95% confidence intervals (CI)." Please explain the meaning of PR in this paper. Is it more accurate to use RR (Relative Risk) instead?
6. In Table 1, in order to conclude that "Children in the index cohort who did not receive MMR1 by 24 months of age were more likely to live with a linked older child who similarly had not received MMR1 by 24 months of age" (page 10, lines 222-224), you need to perform a hypothesis test and present the value of the test statistic and the P-value in detail, this is necessary to make the conclusion more credible. Other characteristics should also perform significance testing.
7. Page 10 line 247-248, should be "of the 11658 index children with no MMR1 receipt within 12-24 month " not "by 24 moths".
8. The manuscript would be benefit from careful proofreading to address grammatical errors and punctuation mistakes. For example in page 4 line 73 "recommends that 95% of the

population are given two MMR doses toachieve herd immunity"

Reviewer 3

Name Gan, Ziming

Affiliation University of Chicago Division of the Physical Sciences, Statistics

Date 04-Jan-2025

COI None

The authors investigated the association between delayed MMR1 vaccination in a child and delays in older children within the same household. The study provides a clear and thorough explanation of data extraction and preprocessing, ensuring reproducibility. The inclusion of a diverse range of variables—spanning individual-, household-, and area-level factors—adds depth to the analysis. Furthermore, the authors adjusted the definition of "timely MMR1 vaccination" to validate their results, effectively demonstrating that households with delays in older children's MMR1 vaccination are more likely to experience delays in younger children's MMR1 vaccination.

1. While the findings are well-supported, the results align closely with expectations and lack novelty. To add value, the authors could develop a predictive model to estimate the probability of timely MMR1 vaccination in children. This could leverage older children's vaccination status as a key predictor while integrating additional variables, such as household-level factors, into a logistic regression or other generative model. Reporting the model's performance and insights derived could make the study more impactful and actionable.

2. The introduction could benefit from additional context and emphasis on the significance of this research.

VERSION 1 - AUTHOR RESPONSE

Reviewer comments	Response
Reviewer 1:<br>The author used Poisson regression to examine the association between MMR1 receipt between ages 12 and 24 months and non-receipt of MMR1 between 12 and 24 months in the oldest child sharing the household. However, Poisson regression requires the assumption of mean and variance equality. Is this assumption	We are grateful to the reviewer for their review of our manuscript and suggestions for review of model diagnostics.<br>On review of the Poisson model diagnostics, we have updated our regression analyses from Poisson to binary logistic. All methods and results have been updated in the manuscript and in the supplementary material.

verified? I would also want to see model diagnoses included.	
The author stated that "The proportion of index children with MMR1 receipt by age 18 months was, as expected, lower than the proportion with MMR1 receipt by age 24 months: 79.2%, 95% CI: 78.9,79.5". I suggest the author report the result using formal language, not connecting all the statistics with a colon at the end of the sentence.	We have corrected the sentence on line 190 to the following:<br>The proportion of index children with MMR1 receipt by age 18 months (79.2%; 95% CI: 78.9,79.5) was, as expected, lower than the proportion with MMR1 receipt by age 24 months (83.6%; 95% CI: 83.3,83.9)
The author stated that "While our study focussed on MMR1 receipt within the UK recommended age range at the time of the study, it is possible that children were vaccinated at older ages". Given the context, I believe the author meant to say "it is possible that children were vaccinated at younger or older ages".	We have changed the sentence on line 195 to the following:<br>It is possible that children were vaccinated outside of the recommended age range of 12 to 24 months.
Reviewer 2:<br>Thanks so much for your article. The primary objective of this study is to examine whether delay in first MMR (MMR1) receipt is associated with sharing a household with an older child with delayed MMR1 receipt. The research design idea is reasonable overall, but there are some details that need to be further modified.	We thank the reviewer for their comments. We have responded to their feedback as follows.
In the study population you included (71,509), the proportion of non-vaccinated individuals is 15.9% (11,658), which is not too rare. Why didn't you consider using logistic regression? What are the advantages of Poisson regression in this situation?	On review of these comments and those from the first reviewer, we have performed model diagnostics and have changed our regression analyses from Poisson to binary logistic. The new results are highlighted in the paper in red, and full analyses are provided in the supplementary file.<br><br>Line 157:<br><br>We estimated the likelihood of MMR1 vaccination between age 12 and 24 months in the linked index cohort using binary logistic regression and estimated odds ratios (OR) and 95% confidence intervals (CI) for those sharing a household with a linked older child with non-receipt of MMR1 between 12 and 24 months of age, before and after adjustment for individual-, household-, and area-level covariates. Covariates with of $p<0.1$ in the univariable logistic regression models were included in a multivariable logistic regression model following a step-wise model selection

	strategy. Variables were retained in the final multivariable model if $p \leq 0.05$.<br><br>Line 202:<br><br>In the unadjusted model, MMR1 receipt between 12 and 24 months of age was less likely among children in the linked index cohort sharing a household with a linked older child with no MMR1 receipt between 12 and 24 months of age (OR: 0.19, 95% CI: 0.18,0.20). The effect size and direction did not change after stepwise introduction of individual-, household-, and area-level covariates resulting in an adjusted OR of 0.20 (0.19,0.21) in the final model (Figure 1; supplementary file 1 Table S3).<br><br>In sensitivity analyses (Figure 2), the proportion of index children with MMR1 receipt between age 12 and 18 months (79.2%; 95% CI: 78.9,79.5) was, as expected, lower than the proportion with MMR1 receipt between 12 and 24 months (83.6%; 95% CI: 83.3,83.9) (supplementary file 1 Table S4). Associations were weaker in sensitivity analyses using this measure as the primary outcome (OR: 0.24; 0.23,0.25) (supplementary 2 file Table S5). By contrast, associations were stronger in sensitivity analyses restricted to households containing index children and linked older cohort children with an age gap of less than five years: OR: 0.14 (0.13,0.15) (supplementary file 1 Table S6). Sensitivity analyses extending the age range for MMR1 receipt to 11-25 months did not change the main findings: OR: 0.18 (0.17,0.19) (supplementary file 1 Table S7).
The Poisson regression model requires that the mean equals the variance of the dependent variable. Does the data exhibit overdispersion?	See above.
I didn't see the goodness-of-fit metrics for the Poisson regression. It would be better to append the results of univariate Poisson regressions and multivariate Poisson regression as supplementary materials to the end of the paper.	Thank you for highlighting the need to investigate the goodness-of-fit metrics. We have now reviewed these and decided to use binary logistic regression instead of Poisson.
Please clarify whether the study population is "eligible to receive MMR1 between 1st January 2020 and 28th February 2020" or "eligible to receive MMR1 between 1st January 2014 and 28th February 2020".	We thank the reviewer for this comment:<br>We can confirm that the study population is eligible to receive MMR1 between 1st January 2014, and 28th February 2020. This has now been amended in the abstract accordingly.
In the abstract's methods section, you mentioned using "Poisson	We have changed our abstract to reflect the new logistic regression. We express our likelihoods as odds ratios.

regression to calculate MMR1 prevalence ratios (PR) and 95% confidence intervals (CI).” Please explain the meaning of PR in this paper. Is it more accurate to use RR (Relative Risk) instead?	
In Table 1, in order to conclude that “Children in the index cohort who did not receive MMR1 by 24 months of age were more likely to live with a linked older child who similarly had not received MMR1 by 24 months of age” (page 10, lines 222-224), you need to perform a hypothesis test and present the value of the test statistic and the P-value in detail, this is necessary to make the conclusion more credible. Other characteristics should also perform significance testing.	Thank you for this helpful comment. Results of the logistic regression models are now presented in the manuscript and supplementary file including 95% confidence intervals.
Page 10 line 247-248, should be “of the 11658 index children with no MMR1 receipt within 12-24 month ” not “by 24 moths”.	We have amended the sentence on line 220 to the following:<br>Of the 11,658 index children with no MMR1 receipt between 12-24 months
The manuscript would be benefit from careful proofreading to address grammatical errors and punctuation mistakes. For example in page 4 line 73 “recommends that 95% of the population are given two MMR doses to achieve herd immunity "	We have amended the sentence on line 35 to the following<br>The World Health Organization (WHO) recommends that 95% of the population are given two MMR doses to achieve herd immunity.<br><br>We have also proof-read and amended the manuscript to make clearer throughout the manuscript that the age interval for MMR1 in the linked index and older child cohorts is 12-24 months and not ‘by 24 months’..
Reviewer 3:<br>The authors investigated the association between delayed MMR1 vaccination in a child and delays in older children within the same household. The study provides a clear and thorough explanation of data extraction and preprocessing, ensuring reproducibility. The inclusion of a diverse range of variables—spanning individual-, household-, and area-level factors—adds depth to the analysis. Furthermore, the authors adjusted the definition of "timely MMR1 vaccination" to validate their results, effectively demonstrating that households with delays in older children's	We thank the reviewer for their positive and detailed review of our paper. We have responded to their comments below.

MMR1 vaccination are more likely to experience delays in younger children's MMR1 vaccination.	
While the findings are well-supported, the results align closely with expectations and lack novelty. To add value, the authors could develop a predictive model to estimate the probability of timely MMR1 vaccination in children. This could leverage older children's vaccination status as a key predictor while integrating additional variables, such as household-level factors, into a logistic regression or other generative model. Reporting the model's performance and insights derived could make the study more impactful and actionable.	We thank the reviewer for these comments.<br><br>Re novelty: While there have been some studies on family concordance of vaccination status, this is to our knowledge the first quantitative study that has examined this using linked health and household data and hence we are reporting novel findings using a novel methodology.<br><br>Re predictive modelling: We have considered the addition of a predictive model into our results which the reviewer considers would make our findings impactful and actionable. Currently a household link is not available in the routine primary health care records in the UK. EMIS practices have a family link but its quality and completeness is unclear and while practitioners can identify people registered at the same address this would be currently an unwieldy and time-consuming approach. Hence currently information about child vaccination status by household is not readily accessible to clinical care teams and predictive models would, in our view, not be feasible to action by primary care staff responsible for delivering MMR1. Further qualitative work would perhaps highlight where in the immunisation pathway information about other household children's vaccination status might be best elicited and how in order to optimise timely household protection with MMR vaccines. Hence at present we do not consider a predictive model would deliver the impact reviewer 3 anticipates although appreciate and agree strongly with the importance of using data to obtain actionable insights.
The introduction could benefit from additional context and emphasis on the significance of this research.	We thank the reviewer for their comment and have added the following paragraph at line 55:<br><br>Identifying factors at a household level can create actionable insights into how services might be tailored to improve receipt of vaccinations.[14] The current pressures on the UK National Health Service have significantly impacted the delivery of vaccinations in primary care- therefore new ways of working to vaccinate the most vulnerable children in a resource-tight setting are needed. [15, 16]

VERSION 2 - REVIEW

Reviewer 1

Name Chen, Xinlei

Affiliation University of Pittsburgh, Department of Biostatistics

Date **11-Apr-2025**

COI

The current version looks good to me.
